# Using neutral, selected, and hitchhiker loci to assess connectivity of marine populations in the genomic era

**DOI:** 10.1111/eva.12288

**Published:** 2015-07-28

**Authors:** Pierre-Alexandre Gagnaire, Thomas Broquet, Didier Aurelle, Frédérique Viard, Ahmed Souissi, François Bonhomme, Sophie Arnaud-Haond, Nicolas Bierne

**Affiliations:** 1 Université de MontpellierMontpellier, France; 2 CNRS – Institut des Sciences de l'Evolution, UMR 5554 UM-CNRS-IRD-EPHE, Station Méditerranéenne de l'Environnement LittoralSète, France; 3 CNRS team Diversity and connectivity of coastal marine landscapes, Station Biologique de RoscoffRoscoff, France; 4 Sorbonne Universités, UPMC Université Paris 06, UMR 7144, Station Biologique de RoscoffRoscoff, France; 5 Aix Marseille Université, CNRS-IRD-Avignon Université, IMBE UMR 7263Marseille, France; 6 Ifremer, UMR “Ecosystèmes Marins Exploités”Sète, France

**Keywords:** connectivity, gene flow, marine conservation, population genomics, population structure

## Abstract

Estimating the rate of exchange of individuals among populations is a central concern to evolutionary ecology and its applications to conservation and management. For instance, the efficiency of protected areas in sustaining locally endangered populations and ecosystems depends on reserve network connectivity. The population genetics theory offers a powerful framework for estimating dispersal distances and migration rates from molecular data. In the marine realm, however, decades of molecular studies have met limited success in inferring genetic connectivity, due to the frequent lack of spatial genetic structure in species exhibiting high fecundity and dispersal capabilities. This is especially true within biogeographic regions bounded by well-known hotspots of genetic differentiation. Here, we provide an overview of the current methods for estimating genetic connectivity using molecular markers and propose several directions for improving existing approaches using large population genomic datasets. We highlight several issues that limit the effectiveness of methods based on neutral markers when there is virtually no genetic differentiation among samples. We then focus on alternative methods based on markers influenced by selection. Although some of these methodologies are still underexplored, our aim was to stimulate new research to test how broadly they are applicable to nonmodel marine species. We argue that the increased ability to apply the concepts of cline analyses will improve dispersal inferences across physical and ecological barriers that reduce connectivity locally. We finally present how neutral markers hitchhiking with selected loci can also provide information about connectivity patterns within apparently well-mixed biogeographic regions. We contend that one of the most promising applications of population genomics is the use of outlier loci to delineate relevant conservation units and related eco-geographic features across which connectivity can be measured.

## Introduction

Inferring population connectivity from molecular data within a population genetic framework can shed light on the evolutionary and ecological processes that shape patterns of genetic diversity (Clobert et al. 2012). Population genetic approaches offer convenient methods to evaluate the rate and scale of dispersal (or migration) when the movement of individuals cannot be assessed by other means such as mark–recapture field experiments. This problem is particularly acute in the marine environment, where the distribution and migratory pathways of organisms are hidden to human eyes underneath the surface of the oceans (Hellberg 2009; Selkoe and Toonen 2011). The potential of genetic methods, illustrated by successful studies in reef species (Selkoe et al. 2010; Puebla et al. 2012), has led to increased expectations for inferring marine connectivity patterns from molecular markers, especially for conservation and management purposes.

**Figure 1:**
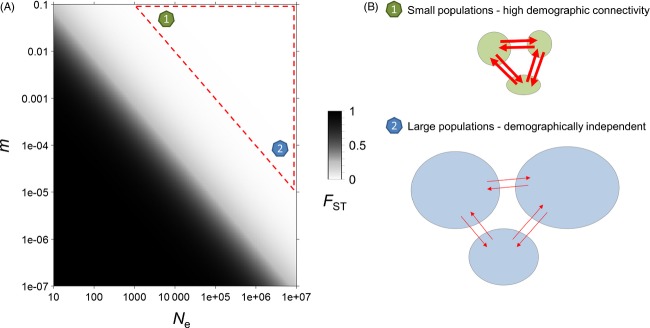
The demographic parameters values behind weak *F*_ST_ values. (A) Because of the nonlinear relationship between *F*_ST_ and *N*_e_*m* in the island model, genetic differentiation (*F*_ST_, in color scale) rapidly shrinks as the per-generation number of migrants (*N*_e_*m*) increases. (B) At equilibrium, weak to null genetic differentiation is expected for small (*N*_e_ = 10^3^) and highly connected (*m* = 10^−1^) populations, but also for large (*N*_e_ = 10^7^) and demographically independent (*m* = 10^−5^) populations.

The majority of marine species, however, display combinations of life history traits (e.g. high fecundity, large population sizes, high dispersal potential often combined to complex life cycles) that produce weak patterns of genetic differentiation or even no differentiation at all (Ward et al. 1994; Waples 1998; Palumbi 2003; Hedgecock et al. 2007). A lack of genetic differentiation may result from a range of situations spanning from nearly complete demographic independence among large-sized populations to the existence of a unique panmictic population (Palumbi 2003; Waples and Gaggiotti 2006; Waples et al. 2008) (Fig. 1). Spatial genetic homogeneity may thus hide a large diversity of scenarios with regard to the contemporary rates of demographic exchanges among groups of individuals inhabiting different parts of a species range. This is of particular concern because the per-generation number of migrants, which is sufficient to lead to apparent genetic panmixia, may not be high enough to ensure demographic connectivity and rescue effects (Waples 1998; Lowe and Allendorf 2010). This discrepancy between the objective of inferring demographic connectivity for conservation biology and management purposes and the limitations inherent to most population genetic approaches has motivated several reviews in the field (Waples and Gaggiotti 2006; Broquet and Petit 2009; Hellberg 2009; Lowe and Allendorf 2010). Our goal here is not to provide a new synthesis of existing methods to infer connectivity, which have been thoroughly addressed in those reviews. We rather aim at considering the new perspectives offered by the increasing number of markers in population genomic studies, with a special focus on the use of loci influenced by selection. The rapid spread of next-generation sequencing (NGS)-based genotyping methods in the molecular ecologists' toolbox has considerably enhanced our ability to identify and characterize genetic variation from population samples (Davey et al. 2011). Still, it remains unclear which approaches will benefit the most from this massive amount of sequence data. One direct benefit of analyzing thousands of markers is an increased precision in measuring genetic differentiation and a higher statistical power to detect small genetic differences among populations (Waples 1998). However, populations with large effective sizes, high migration rates or both may remain virtually undifferentiated, and thus, multiplying neutral markers in such cases may still fail to reveal the current level of demographic connectivity.

Another major achievement offered by NGS approaches is to facilitate the discovery of genetic markers that are influenced by selection (Allendorf et al. 2010; Stapley et al. 2010). These outlier loci can reveal genetic differentiation patterns at the place where neutral markers often remain uninformative, and therefore, it has been suggested that the signal held by outlier loci could be used to delineate locally adapted stocks and redefine conservation units (Nielsen et al. 2009, 2012; Funk et al. 2012). This approach is appealing because selection may be much more efficient than drift in opposing the homogenizing effect of migration, in particular when populations have large effective sizes. However, outlier loci may arise through a wide variety of evolutionary mechanisms apart from local adaptation, which is the primary target of most genome scan studies looking for adaptive variation (Bierne et al. 2013). These evolutionary mechanisms thus need to be identified before using outlier loci to evaluate connectivity.

Allele frequency shifts at outlier loci are expected to be concentrated in particular geographic regions where strong ecological gradients promote local adaptation (Schmidt et al. 2008). Hotspots of genetic differentiation may also arise through the trapping of tension zones by natural barriers to dispersal (Barton 1979a), or through the coupling between exogenous and endogenous reproductive barriers (Bierne et al. 2011). These predictions are corroborated by well-known hotspots of genetic differentiation in the sea (e.g. the Almeria-Oran front, the Siculo-Tunisian strait, Cape Agulhas, Cape Cod, Oresund, Point Conception, among others). However, marine conservation and management issues often require measures of connectivity in areas located outside these particular zones. In a last section, we explore alternative mechanisms that generate disequilibrium at neutral hitchhiker loci even outside the cline of the selected locus itself. These indirect effects of selection can reveal cryptic genetic structure within apparently well-mixed areas. These effects are of two sorts: (i) gradients of introgression (or introgression tails) originating from a geographically distant contact zone (Gagnaire et al. 2011) and (ii) hitchhiking clines that are transiently generated during the propagation of a selective sweep (Bierne 2010). Large population genomic datasets now provide molecular ecologists with the means to use these patterns to study marine connectivity. Therefore, there is a good hope that gathering theoretical background with these new data will further catalyze research in the field.

## Genetic approaches to marine connectivity using neutral markers: successes and limits

Quantitative methods for inferring dispersal with neutral genetic markers fall into two broad categories. One first class of methods looks for the effects of gene flow on the level of genetic differentiation between populations. These methods rely on population genetics models that integrate all relevant evolutionary forces, apart from the effect of mutation which can be neglected for a wide range of migration rates (Box 1). For instance, migration can be indirectly inferred using observations of genetic structure and a model formalizing how gene flow might have produced these observations. The second option is to detect individual dispersal events directly to reconstruct the distribution of dispersal distances, which can be done through genealogy inference (e.g. parentage assignment) or clustering analysis to ascertain population membership of individuals. Not all methods strictly take one of these two routes, but we mention this broad dichotomy here because it gives a good indication of the underlying assumptions, the amount of data required, the nature of the dispersal (or migration) parameter to be estimated, and the spatial and temporal scales over which each method is pertinent and its estimates reliable. The applicability of these methods to three sampling strategies commonly deployed in marine population genetic studies is summarized in Box 2. Below, we briefly describe their broad properties rather than providing an exhaustive catalog, highlighting why both types of dispersal inference methods may be limited in their application for many marine species.

**Box 1: What is the right *F*_ST_ estimator in high gene flow species?**

Since the advent of multi-allelic loci in population genetics, it has been pointed out that the maximum value taken by Wright's *F*_ST_ at a given locus is bounded by its level of genetic diversity so that *F*_max_ ≤ 1 − *H*_S_, where *H*_S_ is the average within-sample diversity (reviewed in Meirmans and Hedrick 2011). Various methods (i.e. estimators aimed at scaling the maximum possible value to 1) have been proposed to correct what is perceived under certain circumstances as a bias, or even a drawback for measuring allelic differentiation between populations (Jost 2008). In the case of bi-allelic loci, all measures (e.g. *D*, *F*', *G*, *θ*; see definitions in Meirmans and Hedrick 2011) give the same equilibrium estimation as Wright's *F*_ST_, which can be transformed into migration rate at migration–drift equilibrium. However, the problem becomes more complex when more than two allelic states occur, and one wishes to take mutation and homoplasy into account. In the island model, 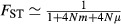, so the relative role of mutation and migration becomes a key issue. In the case of high gene flow species, it is generally admitted that *μ* ≪ *m*. Hence, the main criticism against the use of *F*_ST_ (i.e. *m* = 0, *μ* > 0, which produces a multi-allelic *F*_ST_ estimate tending toward 0 with time despite maximal differentiation and the absence of gene flow) is not justified in such species. On the contrary, *m* ≫ *μ* would imply that the variation detected in one deme is mostly replenished by migration from the metapopulation rather than by locally arisen mutations. Under this assumption, the small differentiation generally observed in most marine species would not be an artifact of using multi-allelic markers, but the consequence of high migration. This has two consequences for our interpretation of genetic variation in high gene flow species: (i) homoplasy is likely to play a very limited role because homoplastic alleles will be almost equally distributed throughout the metapopulation by migration, (ii) high heterozygosity values reflect large metapopulation effective size rather than locally high population size, a pattern that remains true even with relatively low migration between populations.

**Box 2: Neutral methods to infer genetic connectivity**

(A) The distribution of dispersal distances can be estimated in two ways: through the direct detection of discrete dispersal events (blue bars), or the indirect estimation of dispersal parameters like the standard deviation of parent–offspring dispersal distances (*σ*). The mean dispersal distance (*μ*) can be obtained from *σ* by assuming a normal distribution of dispersal distance (blue line). (B) The three sampling strategies commonly used in marine population genetic studies: 1. A geographically subdivided species range is discretely sampled, but some populations are not sampled (gray dotted circle); 2. A continuously distributed species is sampled discretely, and the geographic distance between samples is of the same order as *σ*; 3. Continuous sampling of individuals separated by distances of the same order as *σ*. Colored points are sampled individuals, gray points indicate nonsampled individuals. (C) The information about dispersal that can be obtained from indirect and direct methods for each sampling strategy.

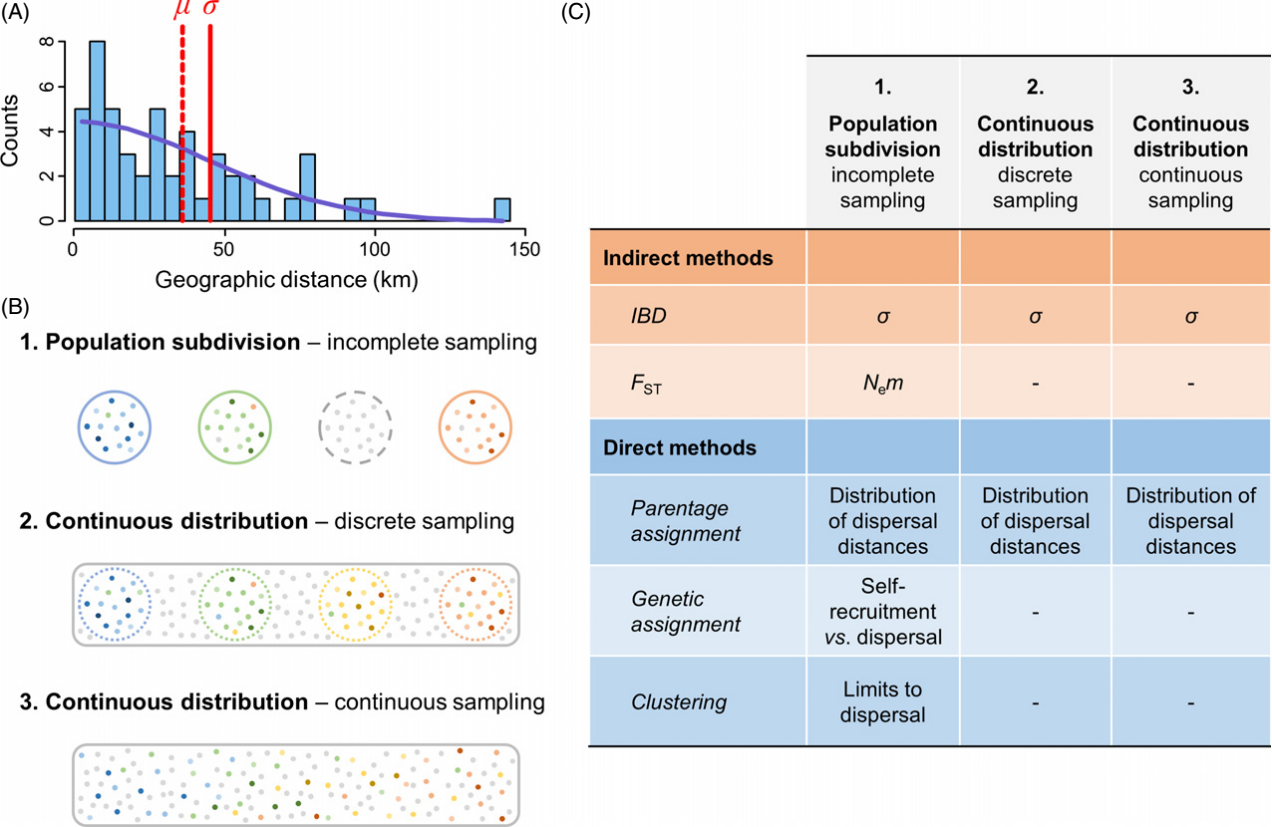

## Inferring genetic connectivity using indirect methods

A representative example of indirect methods is the estimation of dispersal from IBD patterns. The IBD model can be used to estimate dispersal from the increase in genetic differentiation with increasing geographic distances between populations (Rousset 1997) or individuals (Rousset 2000) when dispersal is spatially limited (Box 2). Another example is the estimation of the absolute number of migrants per generation (*N*_e_*m*) from *F*_ST_ in the island model (Wright 1951). Other indirect methods include estimators of *N*_e_*m* or *m* under various extensions of the island model or other more refined population structures (Broquet and Petit 2009). All these methods are associated with a number of generally strong assumptions regarding the structure of populations (e.g. constant and equal size of demes, homogeneous migration, and population density), the life cycle of the species (e.g. nonoverlapping generations, identification of pre- and postdispersal stages and random mating within demes), and the role of each evolutionary force (e.g. negligible effect of selection and negligible or known mutation rate). Model-based methods share at least two important properties. First, a measure of genetic structure never easily translates into an estimate of migration rate (Whitlock and McCauley 1999; Marko and Hart 2011). In particular, a low *F*_ST_ does not necessarily mean that migration is strong as genetic differentiation is influenced by both effective size (*N*_e_) and migration rate (*m*) (Fig. 1). For instance, little dispersal is required to limit the global differentiation in an island model or a stepping-stone migration model (Rousset 2004; and for a recent empirical example: Puebla et al. 2012). Second, dispersal estimates often depend on other known parameters relevant to other evolutionary forces. For instance, the effect of genetic drift must be estimated independently (usually using density estimates) to infer dispersal under the isolation-by-distance model (Pinsky et al. 2010). The main advantage of model-based inference methods is that they require a small amount of data (for the less demanding methods, say about 10 sampling sites with 20 individuals per site). These methods produce estimates of migration rates (*m*) or moments of the distribution of dispersal distances (such as *σ*, the standard deviation of axial dispersal distances, Box 2) which can be difficult to interpret in an ecological context. Finally, such estimates, which have the merit of integrating the effects of evolutionary forces over longer time scales than direct approaches, rely on the questionable hypotheses that dispersal is stable over time and that the migration–drift equilibrium has been reached.

Indirect methods have been applied in a series of case studies in marine organisms. For instance, keeping IBD as a typical example of model-based inference, empirical estimates of *σ* have been reported for a variety of species (Rose et al. 2006; Puebla et al. 2009, 2012; Ledoux et al. 2010; Pinsky et al. 2010). However, such indirect approaches of dispersal can fail on two grounds. First, if genetic drift is too weak to generate population differentiation, then dispersal cannot be inferred using a model that relies on the migration/drift balance. This problem is often encountered in species with extremely large population sizes, such as many marine fishes and invertebrates (DeWoody and Avise 2000; McCusker and Bentzen 2010). For instance, there is no detectable genetic differentiation among populations of the California sea mussel *Mytilus californianus* across 4000 km of its distribution range (Addison et al. 2008). Second, species with large effective population sizes may show patterns of genetic structure that are not at mutation–migration–drift equilibrium. Indirect estimators of dispersal are based on different statistics that evolve at their own speed. Therefore, the rate of approach of equilibrium for a given estimator has to be evaluated to determine whether or not equilibrium is a strong assumption in particular case studies. For that reason, the uncertainty of indirect dispersal estimates due to a possible departure from equilibrium is generally unknown (Pogson et al. 2001).

## Direct estimates of genetic connectivity

In contrast with indirect approaches, the direct detection of migrants through parentage analysis or individual assignment makes much fewer assumptions. For instance, population or parentage assignment methods allow estimating dispersal rates or distances without necessarily relying on demo-genetic models. On the downside, these approaches generally require a great deal of data, very good knowledge of the species distribution, and make the sampling design critical as it must be representative of the postdispersal distribution of individuals (evaluation of long-distance dispersal might be especially difficult due to constraints in the size of the study area). In the case of parentage analysis, an additional constraint stems from the necessity to sample a large fraction of the potential parents. Assignment methods specifically applied to detect immigrants without identifying their origin require less extensive sampling, but their efficiency reduces quickly with decreasing genetic differentiation (but see Gaggiotti et al. 2002). Parental assignments or first-generation migrant tracking methods provide measures of dispersal distances that are relevant to the dispersal episode preceding sampling. Moreover, these methods yield estimates of individual movement rather than gene flow, as immigrants may or may not reproduce locally following dispersal. Finally, although interpreting the results produced by these methods is generally more intuitive than those of indirect approaches, care must be taken regarding the effect of type I errors (i.e. incorrectly identifying a local individual as an immigrant) and unsampled putative parents or source populations (Paetkau et al. 2004; Waples and Gaggiotti 2006). For example, even with high statistical power (no type II error), accepting a 5% type I error for detecting migrants can spuriously increase the estimate of migration rate.

Direct methods have been successfully applied to some marine species. For instance, genetic assignment has yielded useful dispersal information in seals (Gaggiotti et al. 2002), reef fish (Saenz-Agudelo et al. 2011), and corals (Underwood et al. 2007). Similarly, parentage assignment has proven efficient in a number of case studies focusing especially on reef fishes (Jones et al. 2005; Planes et al. 2009; Christie et al. 2010; Almany et al. 2013). Besides a minute type I error, the success of such studies relies upon the fraction of potential source populations or parents that are sampled. These approaches thus require a high-density sampling at a relevant geographic scale, and their application in the marine environment is therefore limited to species with population sizes and distribution ranges that are well documented and small enough for such sampling to be realistic. Although recent studies have shown that larval dispersal oftentimes occurs over smaller spatial scales than previously believed (Swearer et al. 2002; Almany et al. 2007; van der Meer et al. 2012; Puebla et al. 2012), many marine species typically have high fecundity rates, large distribution ranges, and population size (Palumbi 1994). These methods are thus inapplicable to the majority of marine animal species (from invertebrates to pelagic fishes) that have medium to large population sizes, elusive population contours and for which only a minute fraction of the individuals can be sampled for genetic studies.

## Investigating genetic connectivity with clustering methods

When the study species is subdivided into discrete populations, there is a need to first determine the number of populations before evaluating gene flow (Waples 1998). Clustering methods which detect genetic discontinuities and limits to gene flow have been proposed as a way to identify both populations (stocks) and migrants (Pritchard et al. 2000; Broquet et al. 2009). The different clustering approaches (Pritchard et al. 2000; Corander et al. 2003) have their own limits, such as departures from the underlying models (François and Durand 2010). In particular, patterns of isolation by distance may lead to artificial clustering (Schwartz and McKelvey 2009; Blair et al. 2012; Aurelle and Ledoux 2013), and peculiar reproductive systems like partial selfing can induce spurious admixture patterns (Gao et al. 2007). The power of clustering methods generally increases with the amount of genetic differentiation among populations (Latch et al. 2006). For that reason, they are mostly suited to infer genetic connectivity in species with intrinsically or behaviorally limited dispersal abilities and relatively small local population sizes (Ledoux et al. 2010; Wilson and Eigenmann Veraguth 2010; Mokhtar-Jamaï et al. 2011; Perrier et al. 2011; Ansmann et al. 2012; Lukoschek and Shine 2012), which are not representative of the majority of marine species.

## Population genomics using neutral markers for marine connectivity studies: what way forward?

Estimating connectivity from genetic data is a challenging task, which is made even more difficult by the particular life history traits and demographic characteristics of many marine species. More markers may enhance the statistical power of genetic studies and yield more precise estimates of small genetic differentiation values (Patterson et al. 2006), but the signature of dispersal contained in the data may remain intrinsically small or inexistent. In particular, it is not clear whether increasing the number of loci will help in situations where large effective population size keeps genetic structure down, even with restricted migration. As the number of markers rapidly increases, the nonindependence of loci in large population genomic datasets is also becoming another important issue which requires further investigation (Waples 2015).

Despite well-recognized limitations, there is still a good hope that population genomic datasets will improve the usefulness of indirect methods by increasing the power and precision of small genetic differentiation estimates. Although fairly robust estimates of dispersal were already obtained from IBD patterns among populations or discrete geographic samples using tens of markers, greater improvement is expected for methods based on genetic differentiation between individuals (Rousset 2000). This should be achieved through a more accurate estimation of pairwise genetic differentiation between individuals, just like population genomic datasets have improved the inference of relatedness between pairs of individuals for heritability estimation (Visscher et al. 2008). Because the power of isolation-by-distance regression scales with the number of observed pairwise geographic and genetic distances, a continuous sampling of individuals separated by distances in the order of *σ* (Rousset 2000) may be preferable to a discrete sampling of groups of individuals (Box 2).

Analyzing thousands of markers should also increase the power of direct methods, although the type I error issue underlined above is unlikely to be fully resolved even with high power, and sampling requirements cannot be alleviated by intensifying the genetic coverage of each individual. On the other hand, population genomic datasets may also contain useful information on migration events that trace back to several generations in the past. Therefore, extending direct estimates of dispersal beyond the identification of parent–offspring or sibling relationships seems appealing. This should encourage the development of methods that take the full spectrum of relatedness into account.

Whether large datasets will significantly improve the ability of clustering methods to detect existing structure when genetic differentiation is small remains to be tested with recent programs that have been specifically developed for rapidly processing population genomic data (Popescu et al. 2014; Raj et al. 2014). The use of principal component analysis (PCA) methods already proved useful for detecting fine-scale structure between human populations exhibiting low levels of genetic differentiation (Patterson et al. 2006; Novembre et al. 2008). This type of analysis may benefit from the informativeness of rare variants to detect fine-scale population structures (O'Connor et al. 2014), especially in the case of large populations that only exchange few migrants per generation.

Genome-wide polymorphism data that contain information about haplotype phase may open other interesting possibilities for studying connectivity. Immigration followed by successive rounds of sexual reproduction with local residents produces individuals with mixed genetic ancestry. Across generations, the original immigrant chromosomes are progressively broken down by recombination, so that the genome of admixed individuals is composed by a mosaic of segments originating from different ancestral populations (Gompert and Buerkle 2013). The length distribution of such admixture tracts (also called migrant tracts) can be used to infer migration rates between populations (Pool and Nielsen 2009; Gravel 2012). In practice, this approach requires that the ancestry of admixture tracts can be accurately inferred, and this might be possible only when admixture stems from divergent populations. A related approach is based on the analysis of identical genomic segments that are inherited by pairs of individuals. The genomic proportions of long segments that are identical by descent between individuals from the same or different populations are directly related with migration rate (Palamara and Pe'er 2013). Referred to as ‘haplotype sharing’, this approach may be better suited to infer relatively recent migration between populations, although so far it has only been tested using high marker density datasets in species with a high-quality reference genome. These methods are currently under development (Gravel 2012; Liang and Nielsen 2014) and need to be evaluated for their potential to estimate migration in nonmodel species with weakly structured populations contemporarily exchanging migrants. Below, we develop another avenue of research that takes advantage of large population genomic datasets by focusing on genetic markers affected by selection.

## Using selected and hitchhiker loci as an alternative approach to infer marine connectivity

As developed above, the approaches to infer demographic parameters from genetic data classically rely on neutral models that assume a balance between migration and genetic drift (Whitlock and McCauley 1999). As in large populations the effect of drift is very weak, even the most sophisticated methods based on this balance may lack power to infer migration, not to mention that disentangling the effects of *N*_e_ and *m* it is very difficult under these models (Waples 1998; Fig. 1). Alternatively, selection can act as a more efficient antagonistic evolutionary force than drift to counteract the homogenizing effect of migration (Lenormand et al. 1998). As the efficiency of selection scales up with population size, the counterbalancing effect of directional or divergent selection is expected to be greater in marine species with large population sizes (Allendorf et al. 2010). The detection of selected genes has long been a challenging prerequisite, but large marker datasets have considerably enhanced the power of genome scans to identify loci with extreme levels of differentiation (Stapley et al. 2010), the so-called ‘*F*_ST_ outliers’ supposed to be directly or, more probably, indirectly affected by selection (Luikart et al. 2003; Storz 2005). Recent conservation genetic studies have proposed to delineate locally adapted units based on the signal held by outlier loci (Funk et al. 2012; Nielsen et al. 2012), but without providing means to explicitly assess connectivity between such units. Before providing further guidance for using selected markers to infer the rate and scale of dispersal, we consider some of the problems that specifically arise with this category of markers.

## Important concerns related to outlier detection

A common issue encountered in population genomic studies is that the different methods that can be used for identifying *F*_ST_ outliers usually detect only partially overlapping sets of loci. These inconsistencies across methods partly reflect the influence of unknown genetic structure and demographic history on outlier detection tests (for recent reviews, see Narum and Hess 2011; De Mita et al. 2013; De Villemereuil et al. 2014; Lotterhos and Whitlock 2014). In particular, the most commonly used methods for detecting *F*_ST_ outliers have a high rate of false-positive detection under nonequilibrium scenarios (Fraïsse et al. 2014; Lotterhos and Whitlock 2014), hierarchical population genetic structure (Excoffier et al. 2009), and IBD patterns (Meirmans 2012; Fourcade et al. 2013), while suffering at the same time from limited sensitivity (false-negative detection). To circumvent these problems, combining differentiation-based methods with genotype–environment association tests was suggested as a more reliable outlier identification approach (De Villemereuil et al. 2014). In addition, new methods have been developed that are expected to account for correlated ancestry among samples (Excoffier et al. 2009; Bonhomme et al. 2010; Duforet-Frebourg et al. 2014; Foll et al. 2014). However, even if *F*_ST_ outlier tests perform rather well when selection acts on few loci with large effects, they are more seriously challenged by selection acting on many small-effect loci or when the marker loci are loosely linked to the target loci. Because adaptation involving quantitative traits most often evolves through polygenic selection (Pritchard and Di Rienzo 2010), the small changes in allele frequencies resulting from polygenic adaptation may remain below the detection limit of most outlier detection methods (Le Corre and Kremer 2012). In light of recent simulation-based studies that have investigated the performance of outlier tests, it thus appears that outlier candidates should be submitted to validation by combining different statistical approaches, or more directly by comparing allele frequencies before (e.g. in the larval pool) and after (e.g. in juveniles or adults) selective mortality whenever possible (Gagnaire et al. 2012).

An important question that stems from acknowledging the limited power to detect polygenic selection is how to treat the signal held by the most differentiated selected loci, which may have a long, complex, and unanticipated history of divergence, when many others remain undetectable? Apart from the fact that undetected selected loci are expected to bias the neutral-based estimations of connectivity described above, these loci may be more informative than neutral loci for delineating genetic clusters, even if polygenic selection produces small allele frequency changes. Principal component-based analyses combining neutral and selected loci may thus be used as a naive approach to test whether genetic variation is continuously distributed in space, or partitioned into discrete genetic clusters to which individuals can be assigned to estimate the rate and scale of dispersal. Because many species probably match the polygenic selection model, this approach may be appropriate to improve the delineation of discrete genetic clusters in study systems where neutral marker datasets have been uninformative. However, the gain of power offered by large population genomic datasets is difficult to predict and requires further examination using simulated data under different dispersal scenarios.

Another concern when reliable outlier candidates can be identified relates to the nature of the selective effects behind their detection. Several selective mechanisms can increase genetic differentiation above the genome-wide average (Bierne et al. 2013), including underdominance, local and background selection (Charlesworth et al. 1997), but also convergent evolution in response to uniform selection (Ralph and Coop 2010). Importantly, each type of selection can affect neutral loci through linkage, which means that outlier loci could most often result from the indirect effect of selection at other loci. The pervasive effect of selection at linked sites has been well documented in *Drosophila* (e.g. Langley et al. 2012). In such species with large population effective sizes, background selection (Charlesworth et al. 1993) and genetic hitchhiking (Maynard Smith and Haigh 1974) can easily generate correlation between local recombination rates and genetic diversity. Such a correlation has been recently described in the stickleback (Roesti et al. 2012) and the European sea bass (Tine et al. 2014), which confirms that selection at linked sites can also have a dominant effect on genetic diversity in marine species.

In the next sections, we consider geographic patterns generated under various types of selection and provide a guide to infer genetic connectivity using existing and newly developed theoretical frameworks. Applied in local areas with environmental and hydrological singularities, some of these approaches will provide quantitative estimates of dispersal. In other cases, where the effects of selection are less well resolved, genomic data will be helpful to detect genetic discontinuities and provide qualitative assessment of connectivity.

## Estimating dispersal distances using genetic clines

Genome scan studies in marine species have reported several empirical examples of outlier loci exhibiting clinal variation patterns, usually coinciding in space with environmental gradients, ecotones, or boundaries between biogeographic regions (Murray and Hare 2006; Bradbury et al. 2010; Colbeck et al. 2011; Gagnaire et al. 2011; Lamichhaney et al. 2012; Limborg et al. 2012). It is well established that selected markers analyzed in light of cline theory can provide robust estimates of dispersal distances (Barton and Gale 1993; Lenormand et al. 1998; Sotka and Palumbi 2006). Cline shape is basically determined by a balance between migration and selection, which allows under quasi-equilibrium conditions to derive the dispersal parameter in the geographic region of the cline. Empirically inferred dispersal distances may not be precise when only one locus is available and when linkage disequilibrium between selected loci is unknown, but even then they should be of the right order of magnitude (Sotka and Palumbi 2006). Population genomic studies have now the power to detect loci exhibiting clinal variation in species previously believed to be genetically homogeneous, so the potential for discovering new cases of local adaptation clines and cryptic hybrid zones is high (Bierne et al. 2011).

## Using local adaptation clines to infer dispersal

**Figure 2:**
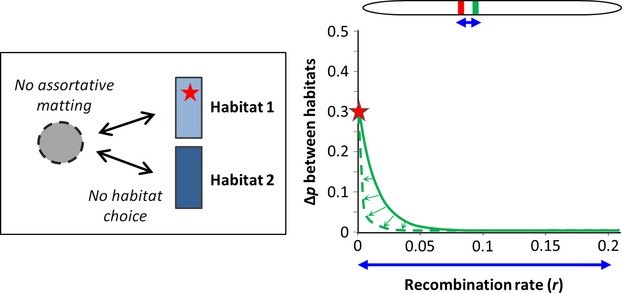
The chromosomal signature of local selection acting on a *de novo* mutation in panmixia. We consider a two habitats Levene's model (Levene 1953) represented in the left box, with random mating (in the dotted circle) and random dispersal (arrows) across two habitats of equal size (rectangles). A new selected mutation (allele *a*, red star) appears in habitat 1 on a haplotype bearing rare neutral variants (in green) at variable recombination distances (the initial frequency is 1/2*N*_e_). The selected mutation has symmetrical antagonistic effects on the fitness of genotypes with respect to habitat (Habitat 1: *ω*_AA_/*ω*_Aa_ = 0.5, *ω*_aa_/*ω*_Aa_ = 2; Habitat 2: *ω*_AA_/*ω*_Aa_ = 2, *ω*_aa_/*ω*_Aa_ = 0.5). At equilibrium, varying selection among genotypes and habitats results in differentiation between habitats at the selected locus (in this example Δ*p* ≈ 0.3). During the progress toward equilibrium, neutral variants hitchhike with the selected allele, transiently producing a narrow chromosomal region where genetic differentiation is increased around the selected locus (green line). As the selected allele progressively recombines away from its haplotypic background, differentiation at neutral alleles rapidly vanishes (green arrows). After a few thousands of generations, differentiation is almost limited to the selected locus (dashed green line).

We refer to local adaptation clines as monogenic clinal variation patterns maintained by a balance between the divergent effects of selection and the homogenizing effects of migration. Such clines occur along environmental gradients or at the frontier between habitats when alternative alleles have antagonistic fitness effects in different environmental conditions (Powers and Place 1978; Koehn et al. 1980). Allele frequencies vary as a sigmoid function of geographic distance (Box 3A) without necessarily reaching fixation if selection cannot purge the inflow of maladapted genotypes (Slatkin 1973). Local adaptation clines can be used to estimate dispersal distance (*σ*) if the selection coefficient (*s*) can be measured, which actually represents a serious challenge to most case studies. However, a measure of selection can sometimes be obtained using experimental populations or genotype frequency comparisons between larvae and adults sampled from the same cohort. By contrast, inferring dispersal from a neutral hitchhiker locus only requires the recombination rate with the selected locus (Box 3A). This can be more readily obtained by studying the signature left by selection in the chromosomal neighborhood of individual outlier loci. For instance, resequencing the region around outliers may help to determine which polymorphism is actually under selection (i.e. the one showing the highest *F*_ST_ value, surrounded by decreasing differentiation on both sides; Box 3A) and provides data to estimate local recombination rates around the selected locus without needing a recombination map (Stumpf and McVean 2003). The chromosomal signature left by local selection in high gene flow species is usually limited to very narrow regions, even when selection acts on *de novo* mutations (Fig. 2). Therefore, high-density genome scans are usually required for efficiently detecting local adaptation loci.

**Box 3: Using selected and hitchhiker loci to infer genetic connectivity**

**Figure 3:**
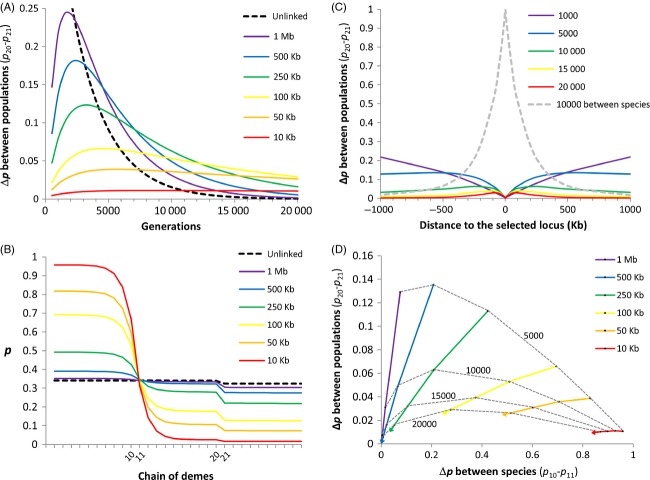
Using the inflow of foreign alleles to reveal within-species connectivity patterns. At generation zero, two partially reproductively isolated species meet on a linear stepping-stone model between demes 10 and 11 and start to exchange genes. The auto-recruitment rate is 1 − *m*, and migration to adjacent demes is *m*/2 (with *m* = 0.5). A weak barrier to gene flow (*m* = 0.01) was set between demes 20 and 21, in the middle of the range of the species localized on the right side. Strong selection (*s* = 0.5) acts against heterozygote genotypes at a reproductive isolation locus, which is linked to neutral markers located at variable recombination distances (from closely linked to unlinked). A recombination rate of 1 cM per Mb was used to convert genetic into physical distances. (A) The step size, calculated as the difference in allele frequency between demes 20 and 21 (Δ*p*), as a function of the number of generations postcontact. (B) Spatial allele frequency patterns after 10 000 generations of introgression showing the frequency step between demes 20 and 21. (C) The step size between demes 20 and 21 as a function of the physical distance to the reproductive isolation locus. (D) The step size between demes 20 and 21 as a function of the difference in allele frequency between species.

Plots show the chromosomal and geographic signatures of selection under four different selective processes. Selected and neutral loci are colored in red and green, respectively. Genetic differentiation (*F*_ST_) along the chromosome is measured between spatial coordinates −500 and 500. (A) A local adaptation cline lying at the frontier between two environments where selection acts in opposite directions (*s* = 0.1, *σ* = 30). The cline width parameter (*w*) is defined as the inverse of the maximum slope at the cline center, and *k* is a coefficient that depends on the selection regime (Slatkin 1973; Nagylaki 1975; Endler 1977; Barton and Gale 1993; Kruuk et al. 1999). A neutral hitchhiker locus with a recombination rate *r* with the selected locus makes a shift (Δ*p*) in the central region of the cline, and an external gradient of allele frequency (∂*p*/∂*x*) directly outside the cline (Barton 1979b). (B) Hybrid zone cline between two partially reproductively isolated populations with selection acting against hybrid genotypes (*s* = 0.5). The amount of linkage disequilibrium (*D*) between selected loci is measured after dispersal at the center of the overlapping clines. (C) A tail of introgression produced by the inflow of foreign alleles entering a subdivided population (see Fig 3 for details). (D) Local connectivity patterns revealed by a global sweep. An unconditionally favorable mutation (*s* = 0.05) appears on the left side of a chain of demes (at an initial frequency of 1/2*N*_e_) and then propagates to the right side from deme to deme (*m* = 0.01), leaving behind a complex allele frequency pattern at a neutral hitchhiking locus (*r* = 0.001). Local connectivity between adjacent demes is transiently revealed by the structure of the neutral hitchhiking locus, as long as gene flow re-homogenizes allele frequencies. The chromosomal signatures of selection can take the form of narrow regions of differentiation (A), large genomic islands (B), or shoulders of differentiation (C and D) centered on the selected loci.

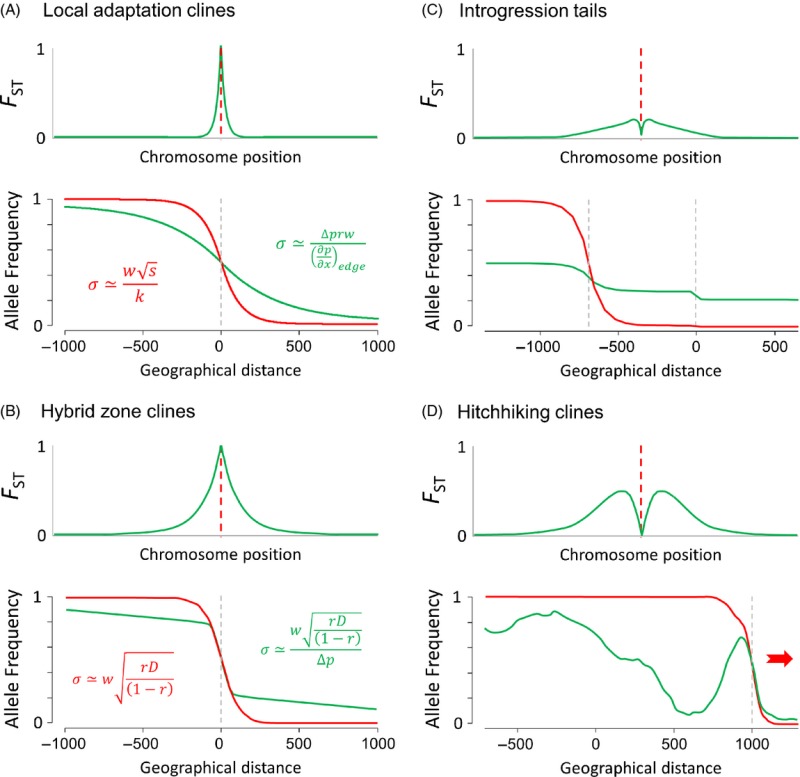

As with parentage assignment methods, the dispersal parameter estimated from local adaptation clines is mostly relevant over short time scales in the geographic area where the shift in allele frequency is observed. However, discordant clines arising in distinct locations in response to spatially uncorrelated selective factors should provide independent local estimates of dispersal across a species range. Local adaptation clines might thus offer valuable alternatives to estimate migration in high gene flow marine species, keeping in mind that the underlying models assume that each cline evolves independently. Therefore, caution must be taken in distinguishing oligogenic from highly polygenic clines. For instance, a high-density genome scan in *Drosophila melanogaster* revealed the existence of several latitudinal clines (Fabian et al. 2012) that geographically overlap with classical clines attributed to local adaptation (e.g. the *Adh* locus, Berry and Kreitman 1993). As for *Drosophila* (Bergland et al. 2015), some classical clines found in marine organisms, such as the *Ldh* cline in the killifish *Fundulus heteroclitus* (Powers and Place 1978), turned out to occur in secondary contact zones (Durand et al. 2009). This suggests that some of the few clinal outliers that were detected trough candidate gene or low-density genome scans may only reflect the emerged part of the iceberg and that polygenic clines and cryptic hybrid zones coinciding with environmental boundaries may be more common than usually believed (Bierne et al. 2011). When significant linkage disequilibrium is detected among clines, the hybrid zone theory offers a more appropriate framework to infer dispersal.

## Using hybrid zone clines to infer dispersal

Many clines evidenced in marine population genetics studies actually result from selection acting at multiple loci, as revealed by the finding of concordant clines in contact zones between hybridizing taxa, that is hybrid zones (Duggins et al. 1995; Bierne et al. 2003; Sotka et al. 2004; Murray and Hare 2006; Zbawicka et al. 2014). In such clines, each locus cumulates the indirect selective effects from other loci (transmitted through linkage disequilibrium) in addition to its own selection coefficient (Barton 1983; Kruuk et al. 1999). The magnitude of indirect effects depends on the amount of linkage disequilibrium and therefore on selection, recombination, and dispersal. The associations among selected alleles in hybrid zones can be used in combination with cline width to infer dispersal (Box 3B, Barton and Gale 1993). As for single locus clines, outlier loci showing concordant clines are not necessarily the actual targets of selection but more likely neutral loci presenting various degrees of linkage with the genes involved in the barrier. Therefore, the shift in allele frequency in the central region of the cline is often much less than 1 for neutral markers, and linkage disequilibrium needs to be corrected for the effect of introgression to estimate dispersal distance (Box 3B).

The cumulative effects of direct selection and indirect selection acting on other loci produce a typical cline shape characterized by a central sigmoid step with two exponential tails of introgression on either side (Barton 1983; Barton and Gale 1993). Allele frequency data collected across a hybrid zone transect can be used to fit a model of cline shape and estimate its parameters, including cline center and width within the narrow region of abrupt change (Szymura and Barton 1986). Hybrid zones analysis programs like *HZAR* provide useful functions for fitting clines along geographic transects (Derryberry et al. 2014).

## Genetic tagging in hybrid zones

A conceptually different approach to estimating connectivity in contact zones is to perform individual genetic assignments to identify migrants. This approach which is similar to the one detailed in the above section (i.e. direct estimates of genetic connectivity) takes advantage of the substantial genetic differences existing between populations or species that are on both sides of the hybrid zone. Minimal dispersal distances can be obtained through the identification of parental genotypes that crossed a hybrid zone and successfully settled in a foreign parental population or species. An even more precise estimation of larval dispersal distance can be made when the source of dispersing larvae is known, as for first-generation hybrids dispersing outside a hybrid zone. Using this strategy, patterns of larval movement among neighboring patches of blue mussels have been examined by measuring realized larval dispersal based on the genetic identification of recently settled juveniles (Gilg and Hilbish 2003). This approach provides an interesting alternative to the measures of dispersal offered by the analysis of genetic clines. In the blue mussel example, both approaches provide comparable estimates: Gilg and Hilbish (2003) found a dispersal distance of 30 km which is in accordance with the 38 km width of the *LAP* cline in Long Island (Lassen and Turano 1978) and the 52 km width of the cline between *M. edulis* and *M. trossulus* in the Oresund (Väinölä and Hvilsom 1991). Because genetic tagging relies on a clear distinction between parental genotypes, introgressed individuals, and real hybrids, individual assignments should be done using the most highly differentiated (and preferentially diagnostic) markers identified in genome scans.

## Using introgression tails to reveal cryptic population structure

Previous methods based on cline width analysis are constrained in their application by the geographic localization of cline centers. However, estimates of population connectivity are often required outside these singular regions, for instance when it is necessary to determine whether there is limited dispersal between populations within areas delimited by ecological or biogeographic boundaries, a relatively common concern for conservation and stock management issues (Allendorf et al. 2010). A potential solution, when the migration–drift equilibrium is not informative, is to search for evidence of spatial structure revealed by introgression (Gagnaire et al. 2011). Introgression may generate gradients (or steps) in allele frequencies along a geographic axis originating at the edge of a contact zone. These tails of introgression may extend to large distances beyond cline centers and can arise for several reasons.

The first one is the free diffusion of neutral alleles following secondary contact between two genetically differentiated populations, or two partially reproductively isolated species. This process creates a transient gradient of introgressing alleles if the rate of introgression is higher or equal to the rate of homogenization within the introgressed population (i.e. the introgression/homogenization rate ratio is ≥1). Importantly, the gradient only appears if dispersal is spatially limited, otherwise spatial homogenization occurs immediately as foreign alleles enter the introgressed population. In order to illustrate how this mechanism can be used to detect a local barrier to dispersal, we simulated a contact zone between two partially reproductively isolated species and the introgression of foreign alleles within one of the two species which is geographically subdivided (Fig. 3). The extent of genetic differentiation within the introgressed species was measured between two populations separated by a weak barrier to gene flow (*m* = 0.01), other adjacent demes being otherwise highly connected in the standard linear stepping-stone model. During a few thousands of generations postcontact, introgression generates a step in allele frequency between the two populations of the introgressed species, and the step then disappears as allele frequencies equilibrate between species (the black dashed line Fig. 3A). Two important properties emerge from these simulations. As the introgression/homogenization rate ratio approaches 1, the magnitude of the frequency step decreases, but the maximum step magnitude is reached later and the step lasts longer. A direct application of these properties is that variable introgression rates among loci provide with the means to detect a weak barrier to gene flow even when introgression has started thousands of generations in the past. For instance, a snapshot taken after 10 000 generations of introgression shows that while the step has completely vanished at freely recombining neutral loci, neutral loci in partial linkage with reproductive isolation loci have retained the signal of differentiation between populations due to their reduced effective migration rate (Fig. 3B). Therefore, differential introgression between parapatric species can be used as a powerful tool to detect cryptic population structure outside the contact zone.

Tails of introgression may be also influenced by selection acting outside the tension zone. In this case, the gradient of allele frequency within the range of the introgressed species may be steepened by a gradient of selection (e.g. an environmental gradient). Because secondary contact zones commonly coincide with environmental gradients (Bierne et al. 2011), introgression tails may be commonly encountered within biogeographic regions separated by environmental boundaries (e.g. the Baltic Sea).

**Figure 4:**
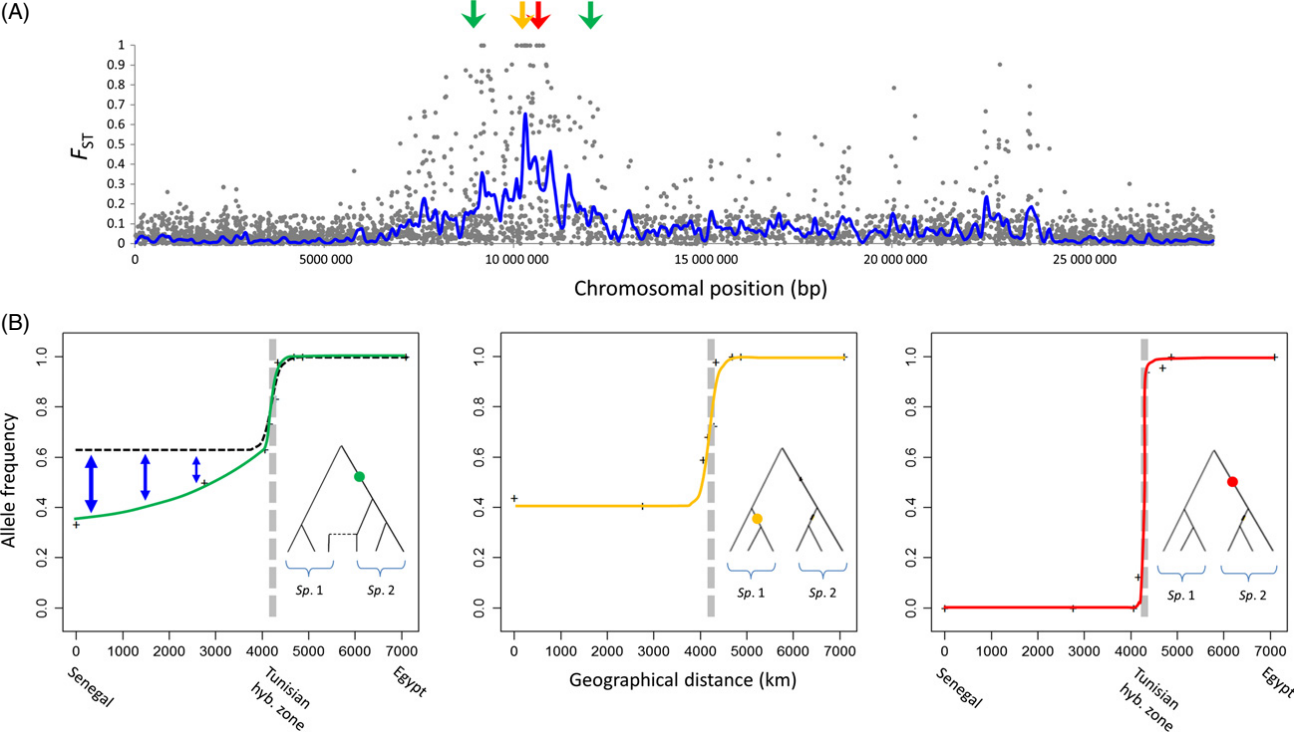
Genomic islands of differentiation and the information therein. (A) A genomic island of differentiation between Atlantic and Mediterranean sea bass lineages (*Dicentrarchus labrax*) on chromosome 7 (RAD-sequencing data from Tine et al. 2014). (B) Geographic clines between two partially reproductively isolated species of sole, *Solea senegalensis* (*Sp*. 1, left side) and *Solea aegyptiaca* (*Sp*. 2, right side) assessed by RAD-Sequencing (A. Souissi, P.-A. Gagnaire, L. Bahri-Sfar, F. Bonhomme, unpublished). Red and orange clines correspond to expectations near reproductive isolation loci (i.e. at the center of a genomic island, where there is no introgression), for a diagnostic locus (red) and a locus only polymorphic in *S. senegalensis* (orange) due to incomplete lineage sorting. The green cline shows a gradient (or a tail) of introgression due to the inflow of *S. aegyptiaca* alleles in the *S. senegalensis* background. At this locus, the shared allele is a consequence of secondary introgression instead of incomplete lineage sorting. Such gradients of introgression are expected to be found at loci showing intermediate degrees of linkage with reproductive isolation loci (i.e. located in the periphery of a genomic island, where introgression is reduced but not zero). Introgression tails may be used to reveal cryptic genetic structure where freely recombining neutral loci remain uninformative (black dashed line), as it is the case in *S. senegalensis*.

These mechanisms show how much it is important to sample not only the whole distribution range of a species but also other divergent populations, or closely related species that live in parapatry or in sympatry before interpreting spatial genetic variation patterns (Gagnaire et al. 2011; Cullingham et al. 2013; Gosset and Bierne 2013). Now that NGS tools begin to reveal genomic islands of differentiation between cryptic species that were previously considered as populations of the same species (Hemmer-Hansen et al. 2013; Karlsen et al. 2013; Tine et al. 2014), polymorphisms located in the periphery of these islands may become a powerful new type of markers to infer connectivity within species, as illustrated in Fig. 4. Importantly, the spatial range of application of genomic-island associated loci could be large if markers are taken at various recombination distances from the central region of a genomic island of differentiation.

## Hitchhiking clines

Another scenario that generates outlier loci happens during the spread of an unconditionally favorable allele in a spatially subdivided population. This process leaves a transient footprint at neutral markers in the chromosomal vicinity of the sweeping allele. When the overall genomic differentiation is low, as it is typically the case in marine species, this process generates an elevated level of differentiation on both sides of the selected locus (Bierne 2010), which corresponds to the locations of sweep shoulders (Schrider et al. 2015). The reason is that recombination progressively breaks the association between the selected locus and the hitchhiker neutral locus, while the sweeping wave propagates. Therefore, the hitchhiking effect is strong at the birthplace of the favorable mutation, while it progressively softens as the wave travels. The effect is stronger for intermediate recombination rates between the selected and the hitchhiker neutral locus, because when linkage is tight, associations remain during the spread of the wave while when linkage is loose the hitchhiking effect is weak right from the beginning. For a similar reason as for the case of introgression clines (Fig. 3C), global hitchhiking therefore generates two shoulders of differentiation on each side of the selected locus on the chromosome. In the deterministic model, the spatial structure generated is a gradient in allele frequency called ‘hitchhiking cline’, but when stochasticity is introduced, for example random genetic drift, the spatial structure can be more complex and sometimes results in nonmonotonic variations in allele frequency (a patchy genetic structure as shown in Box 3D). Detecting the genomic signature of a global sweep requires a high-density screening of the genetic differentiation in the chromosomal neighborhood of the selected locus. Therefore, only few examples that fit the predictions have been studied, with only two cases in marine species (in the blue mussel, Bierne 2010; and the stickleback, Roesti et al. 2014), and some possible cases in highly polymorphic terrestrial species such as maize (Gore et al. 2009; Bierne 2010) and nematodes (Jovelin et al. 2014). By adjusting the global hitchhiking model to the mussel data, it has been possible to estimate the minimal migration rate needed to obtain the observed *F*_ST_ value between the two geographically distant populations of *M. edulis* (Faure et al. 2008) which proved to be surprisingly low (*m* < 10^−8^), as well as the position of the selected locus (−3 kb 5′ of the start codon of the *EF1α* gene), the selection coefficient (*s* = 0.01), and incidentally the local recombination rate of the chromosomal region (*ρ* = 1.7 cM/Mb, Bierne 2010). This result nicely closes the loop of our argumentation by showing how two populations of mussels that are demographically largely independent for thousands of years do not depart from apparent genetic panmixia. Recent analysis based on NGS data (Fraïsse et al. 2015) revealed that deep sampling of the neutral fraction of the genome does not reveal a clear genetic structure between the two populations and that local adaptation is either extremely rare or extremely difficult to evidence (Gosset et al. 2014). Only the indirect effect of selection transiently generated at a linked neutral hitchhiker locus has revealed a sufficiently clear pattern to demonstrate demographic independence.

## Conclusion

Substantial progresses in our understanding of connectivity in nonmodel organism can be achieved with large population genomic datasets. High-density genome scans have reached the power to detect outlying patterns of genetic differentiation at different spatial scales, enabling conservation geneticists to identify genetic differences reflecting restriction to gene flow where classical neutral markers were hitherto most often largely uninformative, as in high gene flow species. The scope of the applications of outlier loci for assessing connectivity patterns in marine species needs further investigations, in particular through gathering a larger set of empirical data. Some of the methodologies that were proposed in this review are still underexplored, and we hope that our work will stimulate new research to test how broadly they are applicable to nonmodel marine species. Although spatially explicit methods are directly applicable to continuously distributed sessile species, selected and hitchhiker loci also have the potential to reveal cryptic genetic structure in migratory species with natal homing (Gagnaire et al. 2011) or feeding migrations. A growing question will be to determine whether all the genetic differences revealed by outlier loci are relevant for conservation and species management. Genome scans will probably confirm the picture of major biogeographic boundaries as hotspots of cryptic genetic structure between populations and partially reproductively isolated species pairs. They may also reveal new and unexpected barriers to gene flow. Such zones are likely to delineate stocks and populations that are important from a conservation point of view. Besides, genome scans may also reveal unusual outlier patterns that are difficult to relate to a clearly identified evolutionary mechanism. The shift to using selected and hitchhiker loci will probably open this can of worms, irrespective to their utility to assess connectivity in the marine realm.

## Abbreviations

Connectivity: The exchange of individuals among populations or subpopulations. Lowe and Allendorf (2010) interestingly distinguished demographic connectivity and genetic connectivity
Demographic connectivity: The relative contribution of net immigration and local recruitment to the population growth rate of a population. Depends both on intrinsic characteristics (survival, reproduction, emigration) of the focal population and the extrinsic contribution of dispersal from other populations
Genetic connectivity: The absolute number of individuals coming into the focal population through immigration from other populations, as measured with genetic data; this may be a measure of individual movement or of gene flow according to the approach used
Dispersal/migration: The movement of individuals between populations
Dispersal/migration rate: The probability of a randomly sampled individual being an immigrant
Genomic islands of differentiation: A region of the genome where genetic differentiation increases above its genomewide average due to the presence of a genetic barrier to gene flow
Hybrid zone: A region where genetically distinct populations are in contact and interbreed
Introgression: The movement of genes between populations or species due to repeated backcrossing
Local adaptation: Higher performance of individuals in the habitat where they were born compared to immigrants
Metapopulation: A group of subpopulations exchanging migrants
Next-generation Sequencing (NGS): High-throughput sequencing techniques (e.g. Illumina sequencing) in contrast to Sanger-based sequencing
Outlier loci: Loci with atypical patterns of genetic differentiation indicative of direct or indirect selection processes
Partially reproductively isolated species: Species that still exchange genes through introgressive hybridization
Population: A group of individuals living in the same habitat and reproducing with each other
Species: A group of individuals which are not interbreeding with other such groups (i.e. *sensu* biological species definition)
Subpopulation, deme: A group of individuals within a population that mate randomly and exchange migrants with other such groups
